# Impact of COVID-19 on Renal Function: Analysis of Acute Kidney Injury Across Three Pandemic Waves

**DOI:** 10.3390/biomedicines13122959

**Published:** 2025-12-01

**Authors:** Mihai Lazar, Cristina Emilia Chitu, Mihaela Cristina Olariu, Ecaterina Constanta Barbu

**Affiliations:** 1 Faculty of Medicine, University of Medicine and Pharmacy Carol Davila, No. 37, Dionisie Lupu Street, Sector 2, 020021 Bucharest, Romania; mihai.lazar@umfcd.ro (M.L.); ecaterina.barbu@umfcd.ro (E.C.B.); 2 National Institute for Infectious Diseases Prof. Dr. Matei Bals, No. 1, Calistrat Grozovici Street, Sector 2, 021105 Bucharest, Romania

**Keywords:** SARS-CoV-2, COVID-19, acute kidney injury, kidney injury, systemic inflammation

## Abstract

**Background/Objectives:** Coronavirus disease 2019 (COVID-19) has emerged as a multisystem disorder, with acute kidney injury (AKI) representing a frequent and severe complication associated with poor outcomes. This study assessed the incidence, risk factors, and outcomes of AKI in patients with severe COVID-19 across three pandemic waves. **Methods:** We retrospectively analyzed 561 patients with severe COVID-19 admitted to a tertiary hospital between March 2020 and December 2021. AKI was defined and staged according to KDIGO 2012 criteria. Demographic, clinical, laboratory, and imaging data were evaluated using univariate and multivariable logistic regression and ROC curve analyses to identify predictors of AKI. **Results:** AKI occurred in 71 patients (12.65%), most frequently during the third wave (40.9%). Stage 1 accounted for 62% of cases, while 23.9% progressed to stage 3 and 10% required dialysis. Compared with patients without AKI, those with AKI had longer hospital stays (15 vs. 11 days), more intense inflammatory responses (CRP 91.7 vs. 63.3 mg/L, *p* = 0.002), and higher mortality (35.2% vs. 10.2%, *p* < 0.001). Multivariable analysis identified elevated serum myoglobin (OR = 1.010, *p* = 0.001), prolonged corticosteroid therapy (OR = 1.096, *p* = 0.035), and lower hemoglobin (OR = 0.375, *p* < 0.001) as independent factors of AKI. **Conclusions:** AKI in severe COVID-19 is multifactorial, reflecting the interplay of systemic inflammation, cytolysis, coagulopathy, and renal microvascular dysfunction. The risk increases with higher myoglobin levels, longer corticosteroid exposure, and lower hemoglobin, highlighting the need for early identification and preventive strategies in high-risk patients.

## 1. Introduction

Since its emergence in late 2019, coronavirus disease 2019 (COVID-19), caused by severe acute respiratory syndrome coronavirus 2 (SARS-CoV-2), has placed an unprecedented burden on healthcare systems worldwide. Beyond its primary respiratory manifestations, COVID-19 has been increasingly recognized as a multisystem disorder, with significant cardiovascular, hematological, digestive, and renal complications [1]. Among these, acute kidney injury (AKI) has emerged as a frequent and severe complication, associated with increased morbidity and mortality [2].

The recent literature shows that the epidemiology and clinical phenotype of COVID-19-associated AKI have evolved over time, with geographic and temporal fluctuations linked to changing dominant SARS-CoV-2 lineages and clinical management patterns. Large temporal analyses report waves of AKI fell after the earliest pandemic peaks but resurged during later variant eras (Delta/Omicron), highlighting that variant-specific disease biology and health-system context can modify AKI burden [3].

The reported incidence of AKI in patients with COVID-19 varies widely, ranging from less than 4% in some early cohorts to over 46% in critically ill populations [2]. This variability likely reflects differences in study populations, geographical settings, definitions of AKI, and healthcare capacities across different phases of the pandemic [3].

Mechanistic and clinical reports from 2023 to 2025 further suggest that later variants have been associated with altered organ-tropism and disease severity profiles, which plausibly influence renal involvement and the spectrum of AKI in hospitalized patients [4].

The pathophysiology of AKI in COVID-19 remains incompletely understood and is the subject of ongoing debate. Proposed mechanisms include direct viral infection of renal cells (via ACE2 receptors), cytokine storm and systemic inflammation, endothelial injury, coagulopathy, hypoxemia, drug-induced nephrotoxicity, and microvascular thrombosis [5,6,7,8,9].

Some authors emphasize that in many patients AKI may result largely from indirect effects (hypovolemia, sepsis, hemodynamic insults), rather than direct viral cytopathy. Others have reported pathologic findings, such as collapsing glomerulopathy, focal acute tubular injury in some cases (especially in patients with APOL1 risk alleles), and functional alterations, particularly in proximal tubules, suggesting direct or variant-specific tropism or damage [10]. The findings suggest that COVID-19-induced systemic inflammation compromises tubular transport mechanisms, resulting in decreased reabsorption of sodium, calcium, and phosphorus and impaired proton excretion [11]. Moreover, studies on post-COVID-19 kidney events demonstrated that AKI is independently associated with both the risk of long-term need for renal replacement therapy and renal function decline [12].

Thus, understanding how these multiple mechanisms interact—and how their relative importance may shift over time (for example, as newer variants, vaccination, or improved care protocols change the milieu)—is an area of active controversy.

Despite the growing scientific data, several gaps and controversies remain. First, the extent to which the incidence and outcomes of COVID-associated AKI have shifted across successive pandemic waves—each characterized by different dominant variants, treatment protocols, hospital burden, and levels of healthcare system preparedness—remains incompletely defined. Second, reported risk factors for AKI in COVID-19 patients showed considerable variation across waves and between different studies. Third, substantial heterogeneity exists across regions and healthcare systems in the reporting of AKI, including differences in diagnostic definitions, availability of baseline creatinine measurements, and the use of renal replacement therapy (RRT). Fourth, comparative data across successive pandemic waves remain limited, and controversies persist regarding the relative contributions of viral pathogenicity, host response, and treatment protocols to the burden of AKI in COVID-19.

The present study addresses these controversies by analyzing the characteristics of AKI in COVID-19 patients, the incidence and staging of AKI in each pandemic wave, and the associated risk factors and outcomes of AKI in COVID-19 patients across three pandemic waves.

Our research hypothesis was that COVID-19-associated AKI is not a static phenomenon but varies across pandemic waves. Specifically, we hypothesized that: (a) the incidence and severity of AKI differ between successive waves (reflecting different variants, treatment protocols, and care-system load), and (b) that the clinical/biochemical correlates of AKI (host response and organ injury markers) also change across waves. Therefore, in addition to characterizing AKI in hospitalized patients with severe COVID-19 and the patterns of clinical/biochemical correlates, we specifically aimed to evaluate whether its incidence and severity differed across the three successive pandemic waves marked by different SARS-CoV-2 lineages.

The novelty of this study resides in the following contributions:

- Providing a comparative analysis across three consecutive pandemic waves, investigating—disease severity, host response, characteristics, incidence, staging, and outcomes of AKI in patients with severe COVID-19;
- Integrating quantitative chest CT metrics (percentage load of consolidation, interstitial, and mixed lung lesions) with classical biochemistry within a unified analytical framework;
- Identifying severity-related factors associated with AKI across the three consecutive pandemic waves;
- Identifying independent factors for AKI using a multivariable model and proposing an exploratory formula to estimate the probability of AKI occurrence in patients with severe COVID-19.

## 2. Materials and Methods

### 2.1. Study Population

We performed a retrospective, observational cohort study at a single tertiary care hospital dedicated exclusively to the treatment of COVID-19 patients. A total of 561 individuals with severe COVID-19 admitted between March 2020 and December 2023 were included.

Patients were stratified into three cohorts based on their admission period, corresponding to the first three pandemic waves [13,14,15]:

- Wave 1: 187 patients admitted between March 2020–January 2021, dominated by the Alpha variant;
- Wave 2: 187 patients admitted between February 2021–June 2021, during the transition period marked by circulation of the Beta variant alongside declining Alpha and emerging Delta;
- Wave 3: 187 patients admitted between July 2021–December 2021, dominated by the Delta variant.

Based on renal outcomes, participants were further classified into two groups: Group A (71 patients with AKI) and Group B (490 patients without AKI).

Eligibility criteria comprised the following:

- Adults (≥18 years) with confirmed SARS-CoV-2 infection, established by either real-time polymerase chain reaction (RT-PCR) or rapid antigen testing;
- Severe forms of the disease;
- Thoracic and abdominal computed tomography (CT) at admission;
- CT image quality—patients with image quality score 4 or 5.

Exclusion criteria were the following:

- Age < 18 years;
- Pregnancy;
- Pre-existing chronic kidney disease;
- Renal malignancy;
- CT image quality score between 1 and 3.

The study protocol conforms to the ethical guidelines of the Declaration of Helsinki and it was approved by the local Ethics Committee of National Institute for Infectious Diseases Prof. Dr. Matei Bals (C10218/15.09.2021).

### 2.2. Definitions

We classified COVID-19 as severe if the patient met at least one of the following: oxygen saturation ≤ 93% on room air, respiratory rate > 30 breaths per minute, PaO_2_/FiO_2_ ratio < 300, or radiologic evidence of lung infiltrates involving more than half of the lung fields [16].

CT image quality was evaluated according to how well the lung parenchyma could be visualized, how sharp vascular borders appeared, and how much blurring was introduced by breathing or patient movement. The following 5-point scale was applied:

1. Poor—parenchymal detail could not be reliably appreciated; vascular margins were not discernible; prominent motion artifact.
2. Fair—lung detail and anatomical boundaries were acceptable but motion artifact was clearly present.
3. Adequate—overall parenchymal and vascular depiction was satisfactory, with only infrequent motion artifact.
4. Good—structures were clearly depicted with very little motion-related degradation.
5. Excellent—sharply parenchymal and vessel visualization with essentially no motion artifact [17].

For positive diagnosis of AKI we used the criteria from the KDIGO (Kidney Disease: Improving Global Outcomes) guidelines [18].

AKI was diagnosed if any of the following criteria was present:

○ Increase in serum creatinine by ≥0.3 mg/dL (≥26.5 µmol/L) within 48 h,
○ Increase in serum creatinine to ≥1.5 times baseline, known or presumed to have occurred within the prior 7 days,
○ Urine output < 0.5 mL/kg/h for ≥6 h.

To ensure consistency across the patient cohort, we applied the second KDIGO criterion—an increase in serum creatinine to ≥1.5 times baseline within the prior 7 days—for AKI diagnosis.

We used AKI Staging (KDIGO) to establish the severity of the renal dysfunction:

Stage 1
  ○ Serum creatinine ↑ ≥ 0.3 mg/dL or 1.5–1.9 × baseline
  ○ Urine output < 0.5 mL/kg/h for 6–12 h
Stage 2
  ○ Serum creatinine ↑ 2.0–2.9 × baseline
  ○ Urine output < 0.5 mL/kg/h for ≥12 h
Stage 3
  ○ Serum creatinine ↑ ≥ 3.0 × baseline or ≥4.0 mg/dL (≥353.6 µmol/L), or initiation of RRT
  ○ Urine output < 0.3 mL/kg/h for ≥24 h or anuria for ≥12 h

To establish the serum creatinine baseline, we assumed a baseline eGFR of 75 mL/min/1.73 m^2^ and back-calculated the corresponding serum creatinine using the Modification of Diet in Renal Disease (MDRD) formula: eGFR (mL/min/1.73 m^2^) = 186 × Scr^−1.154^ × Age^−0.203^ × K, where K = 0.742 for women and K = 1 for men (no Afro-American patients were present in the study group), as suggested by accepted guidelines [18].

Back calculation of serum creatinine resulted in the following formula: Scr = [(186 × Age^−0.203^ × K)/eGFR]^0.866^, where K = 0.742 for women’s and K = 1 for men (no Afro-American patients were present in the study group).

We evaluated the relationship between the measured and estimated creatinine values in patients from Group B and found a strong correlation (R^2^ = 0.813), indicating that the estimated baseline creatinine closely approximated the observed measurements.

### 2.3. Demographic and Biological Parameters

For each patient enrolled in the study, we collected demographic data (sex, age), clinical variables (heart rate, systolic and diastolic pressure, respiratory rate, peripheral oxygen saturation, length of hospitalization), inflammatory markers [C-reactive protein (CRP), serum ferritin, interleukin-1 (IL-1), interleukin-6 (IL-6)], biochemical parameters (alanine aminotransferase, aspartate aminotransferase, creatine kinase, troponin I, lactate dehydrogenase, urea, creatinine, myoglobin, serum glucose, glycated hemoglobin), complete blood count (erythrocytes, leukocytes, lymphocytes, neutrophils), D-dimers levels, plasminogen activator inhibitor-1 (PAI-1), international normalized ratio (INR), prothrombin time (PT), pH, blood gases (O_2_ and CO_2_), and serum electrolytes (sodium, potassium).

### 2.4. CT Examination Protocol

Upon admission, every patient received a chest CT on a 64-detector Definition AS scanner (Siemens Healthcare GmbH, Munich, Germany). The examinations were acquired in helical mode, with CAREDose4D and CARE kV protocols activated to reduce radiation dose.

The acquisition parameters are presented in Table 1 [1].

Lung involvement was quantified using syngoPulmo3D, which reports lesion volume and percentage based on density ranges. We defined four compartments: alveolar (>0 HU), mixed (0 to –200 HU), interstitial (–200 to –800 HU), and normal lung (–800 to –1000 HU) [16,19]. CT datasets were read blinded to clinical information, group assignment, and lab data.

### 2.5. Identification of the Risk Factors/Predictors for AKI in Patients with SARS-CoV-2 Infection

We applied the Mann–Whitney test to evaluate statistically significant differences in continuous variables between Groups A and B, while univariate logistic regression was used for categorical variables. Associations between AKI and admission parameters were examined using Spearman’s rank correlation. The discriminatory capacity of individual risk factors for AKI was further assessed through receiver operating characteristic (ROC) curve analysis. Variables demonstrating statistical significance or clinical relevance were subsequently included in a multivariable logistic regression model to develop prognostic models for AKI.

### 2.6. Statistical Analysis

Statistical analyses were performed using SPSS version 25 (IBM Corp., Armonk, NY, USA). Continuous variables are reported as medians with interquartile ranges, while categorical variables are presented as percentages. Comparisons across the three groups were conducted using ANOVA and binary logistic regression. Associations between clinical parameters and AKI were examined using Spearman’s correlation, and predictive accuracy was evaluated through receiver operating characteristic (ROC) curve analyses. Variables demonstrating clinical importance or statistical significance were entered into multivariable logistic regression models constructed via backward elimination, excluding predictors with *p* > 0.2 according to Wald statistics. Model adequacy was assessed using the Omnibus test, with *p* < 0.05 considered statistically significant [17].

For sample size we considered a confidence level of 95%, a margin of error of 5%, and a population proportion of 50%. We calculated the minimum sample size using the following formula: *n* = [z^2^ × $\hat{p}$ × (1 − $\hat{p}$)]/ε^2^] (*n* = sample size, z = z score, $\hat{p}$ = population proportion, ε = margin of error), resulting in a minimum population of 385 patients. The 561 patients enrolled in our study exceeded the minimum sample requirements, providing more robust data and increasing the reliability of statistical analyses. Based on this sample size, the study is adequately powered to detect moderate effect sizes with sufficient precision for the primary outcomes.

To identify confounders, mediators, and sources of bias, illustrating how corticosteroid duration is confounded by disease severity, demonstrating the presence of immortal-time bias, and providing a transparent causal structure, we performed a directed acyclic graph (DAG) assessment.

To address potential severity-related confounding when evaluating the association between corticosteroid duration and acute kidney injury (AKI), we applied inverse probability of treatment weighting (IPTW). A propensity score (PS) for receiving longer therapy (>10 vs. 1–10 days) was estimated using logistic regression including admission baseline cofounders.

## 3. Results

We identified AKI in 71 cases, accounting for approximately 12.65% of the total study population. The median age of these patients was 56 years [47; 71], with a male-to-female ratio of 1.7:1. This was comparable to patients without AKI, who had a median age of 57 years [46; 68.2] and a slightly lower male-to-female ratio of 1.5:1.

Group A patients exhibited a higher prevalence of comorbidities compared to Group B patients. However, we found no differences with statistical significance between the two groups (Table 2).

Patients in both study groups exhibited comparable heart rate, systolic and diastolic systemic blood pressure, serum glucose, and aminotransferase levels. In contrast, individuals with AKI demonstrated lower blood pH and PCO_2_ values, accompanied by elevated serum potassium concentrations, findings consistent with metabolic acidosis. These patients also exhibited a more pronounced inflammatory response, as reflected by increased CRP levels, elevated total leukocyte counts with neutrophilia, and a higher neutrophil-to-lymphocyte ratio. Markedly elevated lactate dehydrogenase, myoglobin, and creatine kinase concentrations further suggested more extensive cellular injury and cytolysis in Group A. Moreover, patients with AKI showed greater pulmonary involvement, as evidenced by higher proportions of mixed and interstitial lung lesions (Table 3).

Patients in Group A presented a longer median hospital stay compared with those in Group B (15 vs. 11 days), which was associated with a prolonged course of corticosteroid therapy (12 vs. 7 days). The overall mortality rate in the cohort was 13.3% (75 patients), with significantly higher mortality observed in Group A (35.2%) compared to Group B (10.2%). The univariate logistic regression analysis demonstrated that AKI represented an independent risk factor for mortality among patients with severe COVID-19 (Table 4).

We observed a gradual increase in mortality with AKI stage, with a stronger association with mortality for the patients that required hemodialysis (Table 5).

The incidence of acute kidney injury (AKI) among study patients was 12.3% in the first wave, 10.2% in the second, and 15.5% in the third. Although the absolute number of AKI cases was highest during the third wave (29 cases), followed by the first (23 cases) and second waves (19 cases), the 95% confidence intervals for AKI proportions overlapped across waves. This finding suggests that the apparent variation in AKI incidence likely reflects random fluctuation rather than a statistically significant temporal trend (Table 6).

Among affected patients, 62% were classified as stage 1, 14.1% as stage 2, and 23.9% as stage 3 AKI, with 10% of all AKI cases requiring RRT through hemodialysis (Table 7).

Among survivors in Group A, 29 (40.8%) achieved complete renal recovery, 10 (14.1%) had partial recovery, 6 (8.4%) experienced persistent renal dysfunction, and 1 patient (1.4%) remained dialysis-dependent at discharge. These findings indicate that most survivors experienced at least partial improvement in kidney function, whereas a minority had ongoing renal impairment.

The occurrence of AKI demonstrated the strongest positive correlation with serum myoglobin levels, followed by serum potassium, duration of corticosteroid therapy, length of hospital stay, D-dimer levels, serum ferritin, CK, LDH, CRP, neutrophils, WBC, and the extent of pulmonary involvement (both total and interstitial lesions). Conversely, the most significant inverse correlations were observed with blood pH, followed by hemoglobin concentration, platelet count, peripheral oxygen saturation, and blood pCO_2_ (Table 8).

**Figure 1:**
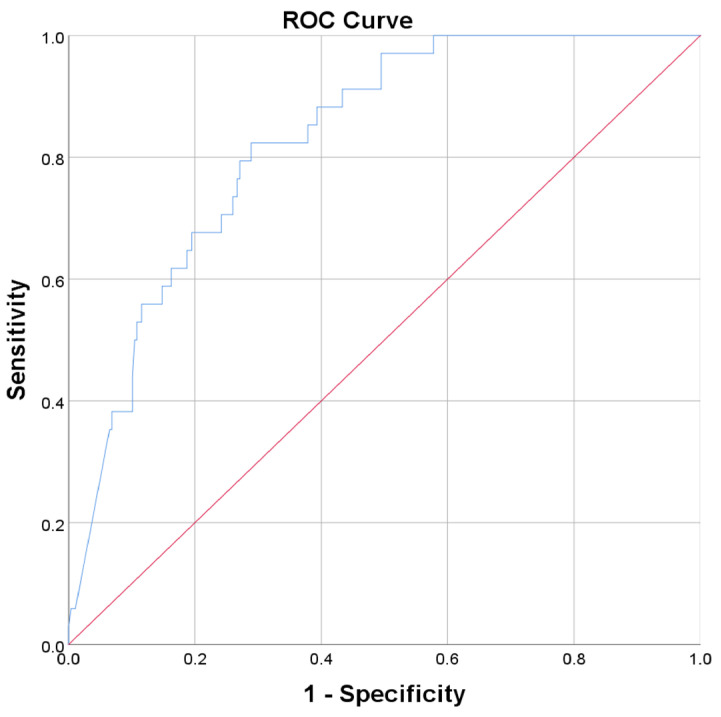
Receiver operating characteristic (ROC) curve (blue) depicting the association of myoglobin with incident AKI (AUC = 0.827, *p* < 0.001). The diagonal reference line is shown in red.

We performed ROC curves analysis for the parameters presented in Table 8, which presented a significant association with AKI, to further evaluate their performance in evaluating the risk of AKI. The highest AUC value was registered for myoglobin (Figure 1) followed by duration of corticosteroid therapy (Table 9).

Although, AKI was significantly associated with blood pH and serum potassium (Table 8), both lower blood pH and elevated potassium may represent consequences of AKI rather than independent predictors. To minimize bias, we excluded them from the predictor analysis.

In COVID-19 care, the duration of both steroid and antiviral therapy generally increases with disease severity and length of hospital stay. Therefore, the observed association between AKI and corticosteroid duration should be interpreted within this context.

During hospitalization, evolving disease severity and prolonged corticosteroid use may both independently and jointly increase the risk of AKI, representing time-varying confounding. Disease progression can directly cause organ injury, precipitating AKI. Prolonged corticosteroid exposure may also raise the risk of secondary infections, sepsis, and nephrotoxic drug use, which act as mediators in the pathway leading to AKI. The directed acyclic graph (DAG) further highlights potential immortal-time bias, as longer corticosteroid exposure requires survival over a longer period—an aspect inherently linked to both “duration of corticosteroid therapy” and “AKI,” since surviving longer increases the likelihood of developing AKI. Baseline variables—including oxygen saturation, FiO_2_, respiratory rate, and total pulmonary lesions—represent disease severity criteria that influence the indication for corticosteroid therapy and confound the relationship between corticosteroid exposure duration and AKI.

**Figure 2:**
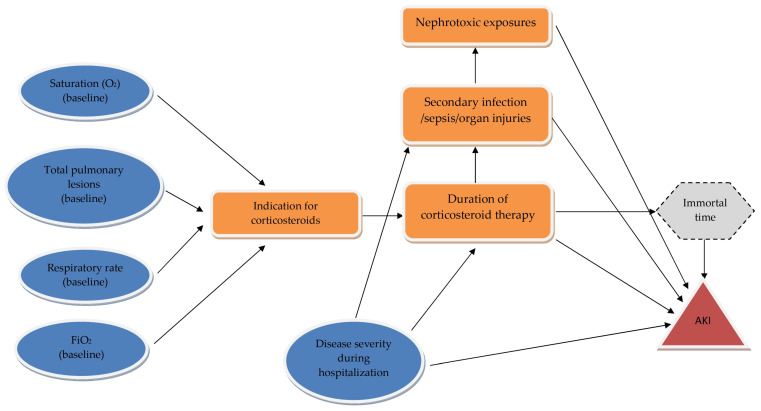
Directed-acyclic-graph to identify minimal adjustment sets. Legend: black arrows—direct assumed causal effects; blue—baseline confounders; orange—mediators; gray—bias source; red—outcome; AKI—acute kidney injury.

Figure 2 presents a DAG illustrating the relationship between corticosteroid therapy and acute kidney injury (AKI).

We applied Inverse Probability of Treatment Weighting (IPTW) to mitigate confounding bias related to disease severity in the analysis of the association between the duration of corticosteroid therapy and acute kidney injury (AKI). The weighted logistic regression model results are presented in Table 10.

The duration of corticosteroid therapy (defined as >10 days vs. 1–10 days) was significantly associated with the development of AKI in the IPTW-adjusted analysis. Patients receiving a longer course of therapy (>10 days) had increased risk of AKI compared to those receiving a shorter course (1–10 days) (Adjusted OR = 1.731, 95% CI: 1.268–2.365, *p* < 0.001).

We performed a multivariable logistic regression model (using the covariates in Table 5 and Table 6) to identify predictors of AKI in SARS-CoV-2 cases. The full model is shown in Table 11. The Omnibus test was highly significant (*p* < 0.001), and the model’s overall classification accuracy was 84.8%.

In the logistic regression model, serum myoglobin (OR = 1.010, 95% CI: 1.004–1.016, *p* = 0.001) and duration of corticosteroid therapy (OR = 1.096, 95% CI: 1.007–1.194, *p* = 0.035) were identified as independent risk factors for the development of AKI. Conversely, hemoglobin concentration demonstrated a protective effect, being inversely associated with the occurrence of AKI (OR = 0.375, 95% CI: 0.229–0.613, *p* < 0.001).

Based on the data in Table 7, we can also calculate the probability of AKI in patients with SARS-CoV-2 infection, using the following exploratory formula:

EXP (7.122 + 0.010 × Myoglobin + 0.092 × duration of corticosteroid therapy − 0.980 × Hemoglobin)/ [1 + EXP (7.122 + 0.010 × Myoglobin + 0.092 × duration of corticosteroid therapy − 0.980 × Hemoglobin)]

## 4. Discussion

### 4.1. Epidemiology and Demographics

Our findings fall within the variable temporal patterns reported in recent multicentre and national studies: while some large series documented a decline in AKI after the earliest waves, later rises during Delta/Omicron periods were also reported, consistent with dynamic, non-linear trends in AKI incidence across the pandemic [10]. Regarding AKI incidence in relation to the three pandemic waves, several cohort studies report higher AKI incidence during Delta-predominant waves, while others show lower AKI risk during Omicron or no clear variant-driven effect once adjustment for vaccination, illness severity, ICU proportion, and therapy is made. Although direct evidence that a specific SARS-CoV-2 variant’s mutations change renal tropism or nephrotoxicity is lacking, the mechanisms proposed (direct renal tropism, inflammation, endothelial injury, rhabdomyolysis) were variant-independent [20].

McAdams et al. compared Omicron, Delta, and other variants, and suggested that severe AKI rates were lower and patients had less severe disease with the Omicron variant [21]. Conversely, other authors reported Omicron variant might be linked to more severe kidney injury in patients diagnosed with AKI at the time of ICU admission, exhibiting a more severe disease course and prolonged hospitalization [22]. Pan et al. reported in their cohort study higher AKI incidence during Delta-predominant periods, with lower severity for Omicron, followed by the Alpha phase. Mechanisms linking variants to renal damage are as follows: direct viral tropism for renal cells (ACE2), dysregulated systemic inflammation, and endothelial injury and microthrombi formation, impairing renal blood flow and causing ischemic damage [23].

Acute kidney injury emerged early in the COVID-19 pandemic as a frequent and prognostically important complication among hospitalized patients. We identified AKI in 71 patients, representing approximately 12.65% of the total study population across the first three waves of the COVID-19 pandemic, a proportion consistent with previously reported data. Reported incidence estimates vary with case-mix and methodology, between 8% in unselected hospitalized series and substantially higher rates (20%) in critically ill subgroups, even ~46% among patients admitted to intensive care units (ICU) [24,25,26].

According to our findings, AKI emerged as an independent risk factor for mortality among individuals with severe COVID-19. In medical data from the literature, advanced age has consistently been associated with greater risk of AKI in COVID-19 cohorts, due to higher prevalence of comorbidities (diabetes, systemic arterial hypertension, cardiovascular disease), and to diminished renal reserve in older adults [24,25]. Several multicenter observational studies also demonstrate that patients who develop AKI have markedly worse short-term outcomes, including substantially increased in-hospital mortality and greater need for RRT; the presence of AKI confers an adjusted several-fold increase in the odds of death across pooled analyses [24]. The mortality rate among patients with concurrent COVID-19 and AKI was 35.2%, which was 2.6-fold higher compared to patients with COVID-19 without AKI.

We obtained a male predominance with a male-to-female ratio of 1.7:1, a median age of patients with COVID-19 and AKI of 56 years and an approximate 10% of patients in this subgroup requiring hemodialysis.

Male gender is over-represented among AKI cases in many cohorts and men with COVID-19 are more likely to progress to severe AKI and to require RRT; proposed mechanisms include sex-dependent differences in immune response, comorbidity burden, and expression of SARS-CoV-2 entry receptors [27]. Nevertheless, interpretation is complicated by confounding (men tended to present with more severe respiratory disease early in the pandemic) and by temporal changes in management; some larger population studies suggest attenuation of the sex effect after adjustment for severity and comorbid conditions.

### 4.2. Pathophysiological Mechanisms and Risk Factors

#### 4.2.1. Direct Viral Infection of Renal Cells

Early autopsy and molecular studies demonstrated SARS-CoV-2 RNA in kidney tissue and virus-like particles in proximal tubular epithelium and podocytes, supporting renal tropism; in some cases replication-competent virus was isolated from post-mortem kidneys [28,29]. Autopsy-based studies have since confirmed SARS-CoV-2 in kidney using RT-PCR, immunohistochemistry, and EM. Hassler et al. detected viral signals in 49% of kidneys by RT-PCR and in 77% by immunofluorescence [30].

Renal susceptibility is mechanistically consistent with high expression of ACE2 and enabling proteases (e.g., TMPRSS2) in proximal tubules and parietal epithelial cells—cellular compartments shown to be permissive for SARS-CoV-2—providing a coherent basis for primary tubular infection and cytopathic injury [31].

Single-molecule fluorescence in situ hybridization and transcriptomic profiling of COVID-19 kidney biopsies show that SARS-CoV-2 infects living renal cells. With infection intensity tracking local ACE2 expression, renal viral detection correlated with worse clinical outcomes, suggesting biological and clinical relevance of direct infection [32]. Infected kidneys display a discrete transcriptomic response involving apoptosis, inflammatory pathways, and profibrotic programs—changes plausibly driving or amplifying AKI [32].

#### 4.2.2. Cytokine Storm and Systemic Inflammation

Direct and indirect amplification of systemic inflammation (“cytokine storm”) is a major proposed driver of COVID-19-associated AKI. Severe SARS-CoV-2 infection induces aberrant innate immune activation with elevated IL-6, IL-1β, TNF-α, and other soluble mediators; this hyperinflammatory state is a potent nonspecific mechanism of multi-organ injury in severe COVID-19 [33,34].

Clinical cohort studies and multicenter analyses have strengthened the link between hyperinflammation and AKI by demonstrating temporal associations between cytokine-release episodes and subsequent deterioration in renal function. In a multicenter retrospective cohort, the occurrence of a defined “cytokine storm” was independently associated with higher odds of AKI after adjustment for baseline comorbidity and respiratory severity, and cytokine increasing frequently preceded AKI onset by days, supporting a causal sequence in the study patients [6,7].

Inflammatory cascades promote endothelial activation, microvascular permeability, coagulation abnormalities, and tissue hypoperfusion linked to tubular injury and impaired filtration [35]. Excessive proinflammatory cytokines can also directly injure renal endothelial and tubular cells, disrupt the filtration barrier, and promote microvascular dysfunction [36]. Activated neutrophils/monocytes release ROS, proteases and NETs, damaging glomerular and peritubular capillaries, promoting microthrombosis, and amplifying endothelial injury [37]. Inflammatory mediators increase vascular permeability (induce interstitial edema), may shift the intrarenal balance toward vasoconstriction (via endothelin, angiotensin II, thromboxane), and dampen vasodilatory pathways (e.g., nitric oxide), thereby reducing renal blood flow and predisposing to ischemic injury [38].

Mechanistic work shows SARS-CoV-2 perturbs pathways (ACE2/angiotensin II, complement, JAK/STAT) that intersect cytokine cascades, amplifying renal susceptibility and providing rationale for targeted anti-inflammatory therapy. Although, cytokine levels in most COVID-19 cohorts are lower than in classical cytokine-release syndromes, sustained moderate hyperinflammation with endothelial/coagulation abnormalities appears sufficient to precipitate clinically relevant AKI [39,40]. Defining patients in whom inflammation predominates (vs. direct viral, ischaemic, thrombotic) remains key for precision use of anti-inflammatory or extracorporeal strategies [41].

Consensus reviews emphasize that systemic inflammation contributes to COVID-19 AKI via (1) cytokine-driven endothelial dysfunction and glycocalyx injury, promoting leukocyte adhesion and microthrombi; (2) hemodynamic instability and capillary leak causing hypoperfusion; and (3) intra-renal inflammatory signaling triggering tubular apoptosis, interstitial inflammation, and early profibrotic programs—consistent with the common pathological finding of acute tubular injury [8].

In our cohort, indicators of systemic inflammation were markedly elevated in patients who developed AKI. Specifically, Group A (AKI) exhibited higher median CRP, ferritin, WBC, neutrophil counts, and a greater neutrophil/lymphocyte ratio. In correlation analyses, CRP, WBC, neutrophils, and the Neu/Ly ratio all showed positive associations with AKI occurrence; their association in univariate and ROC analyses underscores their contributory role in the pathophysiologic environment preceding AKI.

Similarly, retrospective series show elevated CRP, ferritin, and neutrophil counts are more frequent in COVID-19 patients who develop AKI [42,43], and recent data confirm that inflammatory biomarkers correlate with early-onset AKI and non-recovery in critical COVID-19 [44]. Clinically, markedly elevated inflammatory biomarkers should flag a high-risk AKI phenotype, warranting closer renal monitoring, meticulous volume management, avoidance of nephrotoxic drugs, and early supportive measures; anti-inflammatory strategies (e.g., corticosteroids, IL-6 blockade, extracorporeal hemoadsorption) may confer indirect kidney protection, although data remain limited.

#### 4.2.3. Coagulopathy and Anemia: Compromising Renal Microcirculation

Severe COVID-19 is characterized by a distinct thrombo-inflammatory phenotype in which endothelial activation (induced by both viral-induced and host inflammatory signals), complement activation (evidenced by elevated circulating anaphylatoxins and deposition of terminal complement complexes in renal tissue), and platelet activation and dysregulated coagulation converge to promote endothelialitis microthrombi formation, microvascular occlusion involving pulmonary, hepatic, cardiac, and renal capillaries and consequent ischemic injury [8,45,46,47,48]. Early clinicopathologic studies described widespread microvascular fibrin deposition in multiple organs, including the renal cortex, and implicated complement-mediated endothelial injury as a driver of localized thrombotic microangiopathy in severely ill patients [46].

Da Silva et al. found D-dimers were significantly higher in patients with AKI, supporting a microthrombotic contribution to renal injury [49]. Complement activation, platelet aggregation, endothelial activation, and NETs likely reinforce this, producing “no-reflow” and capillary rarefaction, which synergize with hypoxia, inflammation, and tubular injury to trigger AKI [50].

Clinically, COVID-19 coagulopathy presents with markedly elevated D-dimers, often preserved or high fibrinogen, and high rates of macro- and micro-vascular thrombosis that corelate with AKI incidence and severity. Autopsy series and systematic reviews report higher frequency of renal microthrombi in COVID decedents versus controls, supporting a thrombosis–ischemia link [51]. Microvascular thrombosis may cause patchy cortical ischemia, compromise oxygen delivery to proximal tubules, and sustain inflammatory signaling that drives tubular necrosis and interstitial inflammation with hemodynamic instability and hypoxemia as a synergistic cascade [52].

In our cohort, two hematologic/coagulation-related axes stood out as significant correlates of AKI risk: elevated D-dimer levels and decreased hemoglobin concentration. In univariate (Spearman) analysis, D-dimers showed a positive correlation with AKI, while hemoglobin correlated negatively. In ROC analysis, D-dimers achieved an AUC of 0.676 (95% CI 0.601–0.750, *p* < 0.001), and hemoglobin an AUC of 0.728 (95% CI 0.656–0.800, *p* < 0.001). In the multivariable logistic regression model, hemoglobin remained a protective factor (OR = 0.531 per g/dL increase, 95% CI 0.377–0.748, *p* < 0.001), though D-dimers did not remain as an independent predictor when adjusted for myoglobin and serum K. This suggests that anemia’s effect on AKI risk is more independent of confounders in our cohort. The moderate ROC AUC of D-dimers suggests it has utility in stratification but may be best used in conjunction with other markers rather than alone.

Anemia compounds risk by reducing oxygen delivery to vulnerable renal tissue; even with preserved perfusion, low hemoglobin constrains oxygen-carrying capacity, especially in the medulla. Together with microthrombi, anemia narrows the renal oxygen supply–demand reserve and increases susceptibility to ischemic stress—paralleling the non-COVID AKI literature [53]. In COVID-19, anemia may arise from chronic disease, hemolysis, bleeding, dilution from resuscitation, or impaired erythropoiesis—thus it may be both a severity marker and a mechanistic contributor.

Clinically, these relationships imply: (i) in patients with high D-dimers and/or anemia, heightened renal surveillance is warranted; (ii) optimizing oxygen delivery (including transfusion in selected severe anemia) may support renal resilience; (iii) anticoagulation per evolving protocols may mitigate microthrombotic kidney injury, while balancing bleeding risk in anemia; and (iv) avoidance of additive insults (hemodynamic instability, nephrotoxins) is crucial in these hematologic high-risk phenotypes.

#### 4.2.4. Hypoxemia

The kidney is susceptible to decrements in oxygen delivery because tubular solute transport has high metabolic cost, the medulla has low resting perfusion, limited angiogenic reserve, and arteriovenous shunting. These anatomic-physiologic features make the outer medulla and the thick ascending limb of Henle loop vulnerable to reduced oxygen tension, as in COVID-19, due to progressive hypoxemia from pneumonia and acute respiratory distress syndrome (ARDS) [54,55]. Concomitant preservation or increase in tubular oxygen consumption (driven by inflammation and metabolic stress) produces a supply–demand imbalance that precipitates tubular epithelial dysfunction and necrosis—the histologic hallmarks of acute tubular injury frequently seen in COVID-19 AKI [55].

Pulmo-renal crosstalk amplifies this primary mechanism. Severe lung injury provokes systemic inflammation, sympathetic activation, and microcirculatory dysregulation, causing renal vasoconstriction, peritubular capillary rarefaction, and spatially heterogeneous perfusion that further depress medullary oxygenation and induce focal ischemia. Clinically, patients with ARDS and severe hypoxemia have higher AKI incidence and greater renal dysfunction, supporting a causal link between hypoxemia severity/duration and kidney outcome [41,56]. Mechanical ventilation can paradoxically worsen renal oxygenation: high positive end-expiratory pressure (PEEP) and intrathoracic pressures reduce venous return, lower cardiac output, and increase renal venous pressure; the resultant fall in renal perfusion pressure and rise in interstitial pressure impair oxygen diffusion to tubular cells and exacerbate ischemia. Thus, ventilatory management is both a rescue therapy for hypoxemia and a modifiable AKI driver in critical COVID-19 [41,56].

At the cellular level, hypoxia activates hypoxia-inducible factor (HIF) signaling, mitochondrial dysfunction, and reactive oxygen species generation. These responses promote apoptosis, loss of epithelial polarity, and inflammatory recruitment that convert transient oxygen deficits into sustained parenchymal damage. Therefore, experimental and clinical data converge on hypoxemia-driven medullary ischemia as a major—and potentially preventable—mechanism of AKI in severe COVID-19 [54,55].

#### 4.2.5. Metabolic Acidosis and Hyperkalemia in AKI

A first necessary consideration is that acid-base derangement and hyperkalemia may be downstream reflections of evolving AKI rather than pure antecedent risk factors. Early tubular dysfunction can impair H^+^ and K^+^ handling before creatinine rises. However, these derangements also may precede or accelerate AKI rather than only follow it. In severe COVID-19, respiratory acidosis or mixed disorders complicate attribution of acidosis to purely metabolic origins. Nonetheless, lower pCO_2_ and greater oxygenation deficits in our AKI cohort argue against respiratory acidosis as the main driver. Interventional implications are uncertain—whether early bicarbonate therapy or aggressive K^+^ lowering prevent AKI progression remains untested in COVID-19. In standard critical care AKI paradigms, treating severe acidosis and hyperkalemia (bicarbonate, dialysis, K^+^ binders) is considered supportive therapy.

We identified in our study metabolic acidosis (54.9%) and hyperkalemia (29.5%) in the patients who developed AKI. Prior COVID-19 AKI cohorts report hyperkalemia in ~23% of AKI cases, often with coexisting metabolic acidosis [41]. Mathew et al. reported metabolic acidosis in 22.6% of hospitalized COVID-19 patients and found acidosis correlated with worse renal outcomes [57]. Nlandu et al. similarly emphasized that electrolyte disturbances (including hyperkalemia) and acidosis associate with AKI in COVID-19 [58]. Generally, metabolic acidosis reflects tissue hypoperfusion, lactic acid accumulation, impaired renal H^+^ excretion, and reduced buffer reserve. In COVID-19, severe hypoxia, respiratory failure, systemic shock, and multiorgan dysfunction exacerbate lactic acid generation and impair renal compensation. Acidosis may potentiate tubular injury via intracellular acidification, mitochondrial dysfunction, oxidative stress, and proinflammatory pathway activation (e.g., NF-κB), leaving epithelium more vulnerable to ischemia, toxins, and inflammatory mediators [59].

Hyperkalemia often coexists with acidosis via: (1) transcellular K^+^ shift in acidemic states; (2) reduced distal K^+^ secretion due to impaired perfusion, aldosterone resistance, or tubular injury; and (3) diminished K^+^ excretion in already impaired kidney function. In COVID-19, direct viral tubular injury, microvascular injury, or inflammation-mediated tubulopathy may further impair K^+^ excretion. Hyperkalemia itself may propagate injury through tubular cell swelling, mitochondrial dysfunction, and membrane potential disruption [60].

#### 4.2.6. Cytolytic Syndrome and Rhabdomyolysis: Direct Tubular Injury

The pronounced cytolytic syndrome in the patients who developed AKI (Group A), demonstrated by elevated serum values for myoglobin, LDH, and CK, may represent a mechanistic contributor to AKI in COVID-19, beyond being a marker of disease severity. The pathophysiology of rhabdomyolysis-induced AKI (RIAKI) is well established: muscle injury leads to release of intracellular contents into plasma (myoglobin, CK, electrolytes, free iron), and myoglobin plays a central nephrotoxic role. In the kidney tubules, myoglobin precipitates in acidic or volume-depleted states, forming casts and obstructing flow [61]. It also generates reactive oxygen species via Fenton reactions, causes lipid peroxidation, and injures tubular epithelial cells [62], and it can induce renal microvascular vasoconstriction by scavenging NO and triggering vasoconstrictive peptides [63]. Thus SARS-CoV-2–driven muscle injury or generalized cytolysis may be a major source of tubular injury, consistent with autopsy findings of acute tubular necrosis [8]. In COVID-19, multiple reports describe rhabdomyolysis culminating in AKI. Taxbro et al. described severe rhabdomyolysis with AKI by day four of illness, with dark urine, high CK, and rising creatinine [64]. Młynarska et al. discusses how COVID-19 may trigger rhabdomyolysis through direct viral invasion of muscle, immune-mediated muscle injury, or systemic hypoxia and metabolic stress [63]. In the context of COVID-19, the incidence of rhabdomyolysis is increasingly recognized among hospitalized patients, ranging from 0.8% to 48.5%, with the highest rates observed in ICU patients (approximately 50% in some reports—progress to AKI) even though it may often remain underdiagnosed [63,65,66].

In our cohort, the temporal rise in cytolytic markers relative to AKI onset supports the hypothesis that cytolysis may precede or accelerate renal injury, not merely reflect downstream organ failure; the strong OR and ROC supporting a potential causal or contributing role.

However, high CK or myoglobin could also reflect more severe systemic disease (hypoxia, cytokine storm, multiorgan injury), which itself predisposes to AKI via other pathways (hemodynamic instability, inflammation). Thus, defining “pure” rhabdomyolysis as an independent driver from correlated severity may be challenging. Second, subclinical rhabdomyolysis may remain unrecognized, so measured cytolysis may underestimate true burden. Third, interventions targeting myoglobin (e.g., alkalinization of urine, high-flux dialysis, and hemoadsorption) have limited supporting evidence and inherent risks.

From a clinical standpoint, our findings argue for early monitoring of CK, myoglobin, and LDH as part of a renal risk panel in hospitalized COVID-19 patients, especially those with worsening muscular symptoms or unexplained enzyme elevations. In cases of significant cytolysis, aggressive volume resuscitation (while respecting pulmonary limits), urinary alkalinization, and avoidance of additional nephrotoxic drugs may be prudent. In highly selected patients with very high myoglobin loads, extracorporeal modalities aimed to remove them might be considered, though randomized data are lacking.

In summary, the consistent associations in our cohort between elevated myoglobin, CK, LDH, and AKI, combined with mechanistic plausibility and the supporting case literature, support that cytolytic syndrome and rhabdomyolysis represent an important axis for COVID-19-associated AKI. Clinicians should treat marked cytolysis not just as a severity marker but as an actionable red flag for impending renal injury.

#### 4.2.7. Drug-Induced Nephrotoxicity

During the pandemic, polypharmacy (empiric broad-spectrum antibiotics, antivirals, antifungals, adjunctive agents) combined with critical illness physiology (hypoperfusion, inflammation, multiorgan dysfunction) created a high-risk environment in which established nephrotoxins exerted amplified injury. Empirical studies, observational data and expert reviews repeatedly identify aminoglycosides, vancomycin, amphotericin formulations, and some β-lactam/β-lactamase inhibitor combinations as frequent causes of drug-induced AKI (used early in COVID-19 management for suspected superinfection) via direct tubular cytotoxicity, cast formation, and acute interstitial nephritis [8,67].

Remdesivir—the most commonly used direct antiviral in hospitalized COVID-19—was scrutinized because its intravenous formulation contains sulfobutylether-β-cyclodextrin, which accumulates in severe kidney impairment and it contributes to AKI (with risk likely dependent on baseline renal function, cumulative dose, and co-exposures). Pharmacovigilance signals and case series described temporal links to AKI, but controlled analyses have largely not confirmed independent causality after adjustment for illness severity, suggesting that remdesivir can be used with monitoring in many patients with kidney dysfunction [68,69].

Corticosteroids can mitigate cytokine-driven inflammation and protect against immune-mediated tubular damage, yet prolonged or high-dose exposure may predispose to viral reactivations, secondary infections, or sepsis, which themselves may precipitate organ injuries in critically ill patients [70,71]. Steroids also induce marked metabolic stress—hyperglycemia, fluid retention, electrolyte imbalance—each capable of aggravating renal stress so it requires vigilant monitoring of kidney function, glucose, and infectious complications [41]. Hyperglycemia promotes osmotic diuresis and tubular oxidative injury, while sodium and water retention exacerbate renal venous congestion and impair renal perfusion [8]. Observational COVID-19 cohorts suggest a nuanced relationship: steroids may reduce inflammation-driven AKI but simultaneously increase secondary infection–associated AKI, particularly when combined with nephrotoxins. Mechanistically, drug-induced kidney injury in COVID-19 operates through (i) dose-dependent proximal tubular toxicity mediated by intracellular accumulation and mitochondrial injury (aminoglycosides, tenofovir analogs), (ii) crystalluria or intratubular precipitation (some antivirals and high-dose antibiotics), (iii) immune-mediated interstitial nephritis triggered by antibiotics and PPIs, and (iv) tubular cast nephropathy from high-concentration drug–protein complexes (vancomycin-associated casts).

### 4.3. Clinical Trajectory and Outcomes

The clinical trajectory of patients with COVID-19 who develop AKI is consistently more severe, reflecting the convergence of systemic insults, multi-organ dysfunction, and prolonged illness. In our cohort, individuals with AKI experienced significantly longer hospitalizations (median 15 vs. 11 days) and required longer courses of corticosteroid therapy (median 12 vs. 7 days). Pulmonary involvement was also more extensive: patients with AKI had overall higher lung lesion burden (50.6% vs. 43.5%). Mortality was significantly higher in AKI patients (35.2% vs. 10.2%); univariate regression confirmed AKI as an independent risk factor for death, with an odds ratio of 4.75 (95% CI: 2.69–8.38, *p* < 0.001). The literature strongly supports these findings. Multiple observational cohorts and meta-analyses have demonstrated that AKI is a major determinant of adverse outcomes in COVID-19, associated with increased need for intensive care, RRT, and death [72]. Cheng et al. reported that even mild AKI (stage 1) increased mortality risk in hospitalized COVID-19 patients, while stage 3 AKI conferred a particularly severe prognosis [73]. In our research, although most AKI cases were stage 1 (62%), a substantial proportion progressed to stage 2–3 (38%), and 10% required hemodialysis, similar to international reports where dialysis is required in 5–15% of hospitalized COVID-AKI patients, often signaling refractory multiorgan failure [74].

The extended hospital stay in AKI patients in our study likely reflects both the complexity of their illness and the need for intensified monitoring and supportive care. Corticosteroid exposure, longer in the AKI group, may partly reflect more severe pulmonary disease, but prolonged steroid therapy also carries risks of metabolic derangements, secondary infections, and myopathy, which can indirectly worsen renal outcomes. This prolonged trajectory creates a vicious cycle: AKI worsens systemic illness, which in turn perpetuates renal dysfunction. The associations of co-infections (viral, bacterial, fungal, and parasitic) should also be considered, as these are linked to a more severe form of the disease, resulting in prolonged hospitalization and more complications [75].

Greater lung involvement, as observed in our AKI group, can worsen hypoxemia and systemic inflammation, which in turn amplify renal vulnerability. The concept of “lung–kidney cross-talk” has been highlighted in COVID-19, where respiratory failure and systemic inflammation mutually aggravate renal perfusion and function [72]. In our patients, lower oxygen saturation and higher FiO_2_ requirements were consistent with this inter-organ interplay.

Mortality in COVID-19 is multifactorial, but AKI clearly acts as both a marker of severity and an independent contributor. Inflammation, microvascular thrombosis, and metabolic derangements converge in AKI, accelerating multiorgan failure. The 35.2% mortality in our AKI cohort is consistent with international series reporting mortality between 30 and 50% in COVID-19 patients requiring dialysis or presenting with severe AKI [76].

Although only a limited number of studies have examined the impact of COVID-19 vaccination among those who subsequently develop AKI, the findings indicate a significant protective effect. Vaccination was independently associated with reduced mortality and shorter hospital stays among patients with COVID-19-related AKI due to lower rate of respiratory complications and cardiovascular events (consecutive to decreased inflammatory and immune responses, microvascular thrombi, and hemodynamic effects for COVID-19 vaccinated patients) [77].

Our findings confirm that AKI is not an isolated complication but a central determinant of clinical trajectory in COVID-19 with prolonged length of hospital stay, increased treatment complexity, greater pulmonary injury, and higher mortality. Early recognition and prevention of AKI, through rigorous monitoring of renal function, judicious fluid and hemodynamic management, and mitigation of inflammatory and microvascular injury remain essential to improving outcomes.

*Study limitations*. First, this research was performed in a single tertiary center, and the findings may not be fully generalizable to different healthcare systems, populations, or pandemic phases. Second, its retrospective design limits causal inference. Third, baseline serum creatinine was back-calculated assuming an eGFR of 75 mL/min/1.73 m^2^, which may misclassify some patients with unrecognized pre-existing renal dysfunction. Fourth, follow-up was limited to the in-hospital period, so long-term renal recovery and post-discharge outcomes could not be assessed. Finally, residual confounding by unmeasured variables (e.g., detailed drug dosing, timing of therapies, or viral genomic characterization) cannot be excluded.

Despite these limitations, the strict inclusion criteria, uniform CT-quantification protocol, and standardized data collection across the three consecutive waves strengthen the internal validity of our findings.

## 5. Conclusions

Acute kidney injury represented a frequent and severe complication in patients hospitalized with COVID-19, affecting approximately 12.6% of our cohort. Although AKI was associated with substantial in-hospital mortality (35.2%), most survivors experienced renal recovery, reflecting the predominance of mild (stage 1) AKI. The absolute number of AKI cases was highest during the third wave (15.5%), followed by the first (12.3%) and second one (10.2%), but the apparent variation likely reflects random fluctuation rather than a statistically significant temporal trend.

AKI occurrence in severe COVID-19 was multifactorial, with elevated myoglobin and prolonged corticosteroid therapy emerging as factors independently associated with AKI, while higher hemoglobin exerted a protective effect. Cytolytic syndrome, coagulopathy, hyperinflammation, and extensive pulmonary involvement further amplified risk, highlighting the interplay between systemic inflammation, tissue injury, and renal microcirculatory compromise.

Our findings support vigilant biochemical monitoring (including cytolysis markers), careful duration of corticosteroid therapy, and early supportive interventions to prevent AKI in high-risk patients. An integrated, multidisciplinary approach that combines renal, pulmonary, and critical care perspectives remains essential to mitigate the burden of AKI and its associated morbidity and mortality in severe COVID-19.

## Figures and Tables

**Table 1:** Technical parameters of CT scans.

Parameters	CT Scan Values
Slice thickness (mm)	3
Reconstruction thickness (mm)	1.5
Colimmation	1.2
Reference mAs	250
Reference kV	120
Rotation time (s)	0.5
Pitch	0.35
FOV	Both lungs/thorax and superior abdomen included
Reconstruction kernels	H31f for mediastinum and superior abdomen; H60f for lung

Abbreviations: mAs-milliangstroms, kV—kilovolts, FOV—field of view.

**Table 2:** Comorbidities in COVID-19 patients with and without AKI.

Comorbidity	Group A (*n*, %)	Group B (*n*, %)	*p*-Value
Obesity	18 (25.3%)	148 (30.2%)	0.400
Type 2 diabetes mellitus type-2	10 (14.1%)	49 (10%)	0.297
Systemic arterial hypertension	24 (33.8%)	128 (26.1%)	0.175
Congestive heart failure	2 (2.8%)	16 (3.2%)	0.841
Peripheral vascular disease	3 (4.2%)	12 (2.4%)	0.392
Chronic obstructive pulmonary disease	6 (8.4%)	31 (6.3%)	0.502
Chronic viral hepatitis	3 (4.2%)	15 (3%)	0.605
Personal history of neoplasia	2 (2.8%)	12 (2.4%)	0.853
Personal history of ischemic stroke	5 (7%)	19 (3.8%)	0.225
Dementia	2 (2.8%)	6 (1.2%)	0.304
Personal history of peptic ulcer	1 (1.4%)	5 (1%)	0.767

**Table 3:** Clinical, biological, and imaging characteristics in Group A and Group B patients.

Clinical, Biological, and Imaging Characteristics	Group A (Median, Q1, Q3)	Group B (Median, Q1, Q3)	*p*-Value
Heart rate (beats/min)	80 [72; 96]	84 [74; 94]	0.249
Systolic pressure	130 [114; 144]	128 [117; 137]	0.428
Diastolic pressure	76 [65; 82]	79 [70; 84]	0.066
Respiratory rate (breaths/minute)	22 [20; 27]	22 [20; 28]	0.871
Saturation (O_2_) %	95 [91; 97]	96 [94; 98]	0.001
FiO_2_ (%)	60 [40; 60]	55 [40; 60]	0.037
pO_2_ (mmHg)	79 [62; 108]	84 [70; 104]	0.222
pCO_2_ (mmHg)	34 [30; 40]	37 [34; 40]	0.004
pH	7.33 [7.31; 7.37]	7.4 [7.37; 7.43]	<0.001
Serum Na (mEq/L)	137 [136; 139]	138 [136; 140]	0.321
Serum K (mEq/L)	4.8 [4.3; 5.3]	4.2 [3.8; 4.5]	<0.001
CRP (mg/L)	91.7 [43.9; 148.2]	63.3 [21.8; 122.7]	0.002
Length of hospital stay (days)	15 [11; 18]	11 [6; 17]	<0.001
Duration of antiviral treatment (days)	5 [4; 5]	5 [3; 6]	0.692
Duration of corticosteroid therapy (days)	12 [10; 15]	7 [3; 12]	<0.001
Serum glucose (mg/dL)	97 [85; 109]	96 [87.5; 107.5]	0.977
HbA1c %	5.51 [5.13; 5.93]	5.39 [5.11; 5.80]	0.294
LDH (U/L)	454 [253; 663.5]	350.5 [277.7; 433]	0.002
AST (U/L)	53 [32; 112]	46 [34; 63]	0.060
ALT (U/L)	44 [24; 108]	44 [30; 74]	0.857
CK (U/L)	167.5 [69; 309]	86 [55; 162]	<0.001
CK-MB (U/L)	12.5 [6; 16]	10 [6; 15]	0.190
Myoglobin (ng/mL)	308.5 [197; 400]	120.2 [92.6; 207.2]	<0.001
Serum urea (mg/dL)	39 [34; 48]	34 [27; 43.5]	<0.001
Serum creatinine (mg/dL)	1.8 [1.5; 3.1]	0.8 [0.7; 1]	<0.001
Serum ferritin(ng/mL)	1431 [928.5; 1650]	1006 [381; 1504]	0.001
IL6 (pg/mL)	48 [6.1; 194.6]	22.5 [2.4; 151.7]	0.394
PAI-1 (ng/mL)	398 [154; 738]	501 [294; 673.2]	0.421
Hemoglobin (g/dL)	12.4 [9.6; 13.6]	13.9 [12.8; 14.6]	<0.001
WBC (×10^3^/µL)	8.7 [6; 14.6]	6.6 [5.4; 9]	0.001
Lymphocytes (×10^3^/µL)	0.7 [0.5; 1.2]	0.9 [0.7; 1.2]	0.036
Neutrophils (×10^3^/µL)	7 [4.5; 13.3]	5.2 [3.8; 7.5]	<0.001
Neu/Ly ratio	9.3 [4.8; 17.1]	5.6 [3.3; 10]	<0.001
Platelets (×10^3^/µL)	196.5 [132.7; 252.2]	221.5 [183; 300]	<0.001
D-dimers (ng/mL)	484 [216; 1141]	246 [164; 409]	<0.001
INR	1.06 [1; 1.19]	1.07 [1; 1.14]	0.752
PT (s)	12.9 [11.9; 14.1]	12.9 [12.1; 13.8]	0.721
Number of pulmonary lobes involved	5 [5; 5]	5 [5; 5]	0.452
Consolidation (% from total lung volume)	2 [1.5; 3.1]	1.6 [1.2; 2.3]	<0.001
Mixed lesions (% from total lung volume)	2.9 [2.1; 4.8]	2.2 [1.5; 3.9]	0.002
Interstitial lesions (% from total lung volume)	45.5 [36.8; 55.5]	39 [32.7; 49.6]	0.001
Normal pulmonary densities (% from total lung volume)	49.4 [37.9; 58.1]	56.4 [42.5; 64.2]	0.001
Total pulmonary lesions (% from total lung volume)	50.6 [41.9; 62.1]	43.5 [35.8; 57.5]	0.001

Abbreviations: ALT—alanine transaminase; AST—aspartate transaminase; CK—creatine kinase; CRP—C-reactive protein; FiO_2_—fraction of inspired oxygen; HbA1c—glycated hemoglobin; IL—interleukin; INR—international normalized ratio; K—potassium; LDH—lactate dehydrogenase; Ly—lymphocytes; Na—natrium/sodium; Neu—neutrophils; pO_2_—partial pressure of oxygen; pCO_2_—partial pressure of carbon dioxide; PT—prothrombin time; WBC—white blood cells.

**Table 4:** Univariate logistic regression model for mortality in study group.

Variable	B	S.E.	Wald	*p*	OR	95% CI for OR	
						Lower	Upper
AKI	1.558	0.290	28.893	<0.001	4.750	2.691	8.384
Constant	−2.168	0.149	210.865	<0.001			

Abbreviations: AKI—acute kidney injury; CI—confidence interval; OR—odds ratio; S.E.—standard error.

**Table 5:** Characteristics of mortality in Group A patients.

Parameter	Survivors (n)	Non-Survivors (n)	Total (n)	Mortality (%)	OR (vs. No AKI)	95% CI	*p*-Value
Patients without AKI	440	50	490	10.2%	1	—	—
Stage 1	31	13	44	29.5%	3.68	1.81–7.48	<0.001
Stage 2	6	4	10	40%	5.85	1.59–21.4	0.006
Stage 3	9	8	17	47.1%	14.0	6.03–32.5	<0.001
Hemodialysis	1	6	7	85.7%	52.6	6.17–445	<0.001
AKI	46	25	71	35.2%	—	—	—

Abbreviations: AKI—acute kidney injury; CI—confidence interval; n—number of patients; OR—odds ratio.

**Table 6:** Proportion of AKI cases among the three waves.

Wave	AKI Cases (*n*)	Total Patients (*n*)	Proportion	95% CI
1	23	187	12.3%	7.6–17%
2	19	187	10.2%	5.8–14.5%
3	29	187	15.5%	10.3–20.7%

Abbreviations: AKI—acute kidney injury; CI—confidence interval.

**Table 7:** Characteristics of AKI in Group A patients.

Parameter	Patients in Wave 1	Patients in Wave 2	Patients in Wave 3
AKI	23 (32.4%)	19 (26.7%)	29 (40.9%)
Stage 1	16 (22.5%)	11 (15.5%)	17 (23.9%)
Stage 2	3 (4.2%)	2 (2.8%)	5 (7%)
Stage 3	4 (5.7%)	6 (8.4%)	7 (10%)
Hemodialysis	2 (2.8%)	2 (2.8%)	3 (4.2%)

Abbreviations: AKI—acute kidney injury.

**Table 8:** Correlations of clinical, biological, and imaging characteristics with AKI.

Clinical, Biological, and Imaging Characteristics	Spearman’s Rho	*p*-Value
Heart rate (beats/min)	−0.049	0.249
Systolic pressure	0.034	0.429
Diastolic pressure	−0.078	0.066
Respiratory rate (breaths/minute)	0.007	0.732
Saturation (O_2_) %	−0.142	0.001
FiO_2_ (%)	0.088	0.037
pO_2_ (mmHg)	−0.055	0.223
pCO_2_ (mmHg)	−0.135	0.004
pH	−0.390	<0.001
Serum Na (mEq/L)	−0.042	0.322
Serum K (mEq/L)	0.331	<0.001
CRP (mg/L)	0.135	0.002
Length of hospital stay (days)	0.219	<0.001
Duration of antiviral treatment (days)	0.018	0.692
Duration of corticosteroid therapy (days)	0.315	<0.001
Serum glucose (mg/dL)	0.001	0.977
HbA1c %	0.052	0.293
LDH (U/L)	0.135	0.002
AST (U/L)	0.083	0.060
ALT (U/L)	−0.004	0.917
CK (U/L)	0.157	<0.001
CK-MB (U/L)	0.057	0.190
Myoglobin (ng/mL)	0.354	<0.001
Serum ferritin(ng/mL)	0.168	0.001
IL6 (pg/mL)	0.052	0.395
PAI-1 (ng/mL)	−0.062	0.423
Hemoglobin (g/dL)	−0.263	<0.001
WBC (×10^3^/µL)	0.148	<0.001
Lymphocytes (×10^3^/µL)	−0.089	0.035
Neutrophils (×10^3^/µL)	0.164	<0.001
Neu/Ly ratio	0.168	<0.001
Platelets (×10^3^/µL)	−0.154	<0.001
D-dimers (ng/mL)	0.204	<0.001
INR	0.014	0.752
PT (s)	0.015	0.721
Number of pulmonary lobes involved	−0.039	0.356
Consolidation (% from total lung volume)	0.161	<0.001
Mixed lesions (% from total lung volume)	0.130	0.002
Interstitial lesions (% from total lung volume)	0.145	0.001
Normal pulmonary densities (% from total lung volume)	−0.144	0.001
Total pulmonary lesions (% from total lung volume)	0.144	0.001

Abbreviations: ALT—alanine transaminase; AST—aspartate transaminase; CK—creatine kinase; CRP—C-reactive protein; FiO_2_—fraction of inspired oxygen; HbA1c—glycated hemoglobin; IL—interleukin; INR—international normalized ratio; K—potassium; LDH—lactate dehydrogenase; Ly—lymphocytes; Na—natrium/sodium; Neu—neutrophils; pO_2_—partial pressure of oxygen; pCO_2_—partial pressure of carbon dioxide; PT—prothrombin time; WBC—white blood cells.

**Table 9:** ROC curve analysis for parameters associated with AKI.

Predictor	AUC	Std Error	*p*-Value	95% CI	
				Lower Bound	Upper Bound
Saturation (O_2_)	0.623	0.036	0.001	0.551	0.694
FiO_2_ (%)	0.574	0.039	0.044	0.497	0.651
pCO_2_ (mmHg)	0.612	0.047	0.005	0.521	0.704
CRP (mg/L)	0.619	0.032	0.002	0.555	0.682
Length of hospital stay (days)	0.678	0.026	<0.001	0.627	0.730
Duration of corticosteroid therapy (days)	0.764	0.022	<0.001	0.720	0.808
LDH (U/L)	0.618	0.046	0.002	0.529	0.707
CK (U/L)	0.644	0.040	<0.001	0.566	0.723
Myoglobin (ng/mL)	0.827	0.031	<0.001	0.767	0.888
Serum ferritin(ng/mL)	0.657	0.041	0.001	0.577	0.738
Hemoglobin (g/dL)	0.728	0.037	<0.001	0.656	0.800
WBC (×10^3^/µL)	0.628	0.041	0.001	0.548	0.709
Lymphocytes (×10^3^/µL)	0.577	0.040	0.036	0.498	0.657
Neutrophils (×10^3^/µL)	0.643	0.039	<0.001	0.565	0.720
Neu/Ly ratio	0.646	0.037	<0.001	0.573	0.718
Platelets (×10^3^/µL)	0.634	0.037	<0.001	0.561	0.707
D-dimers (ng/mL)	0.676	0.038	<0.001	0.601	0.750
Consolidation (% from total lung volume)	0.640	0.033	<0.001	0.575	0.704
Mixed lesions (% from total lung volume)	0.612	0.032	0.002	0.551	0.674
Interstitial lesions (% from total lung volume)	0.626	0.032	0.001	0.562	0.689
Total pulmonary lesions (% from total lung volume)	0.625	0.031	0.001	0.563	0.687

Abbreviations: AUC—area under curve; CI—confidence interval; CK—creatine kinase; CRP—C-reactive protein; FiO_2_—fraction of inspired oxygen; K—potassium; LDH—lactate dehydrogenase; Ly—lymphocytes; Neu—neutrophils; pCO_2_—partial pressure of carbon dioxide; WBC—white blood cells.

**Table 10:** Association between duration of corticosteroid therapy and AKI in the IPTW-adjusted analysis.

Variable	B	S.E.	Wald	*p*	OR	95% CI for OR	
						Lower	Upper
Duration of corticosteroid therapy	0.549	0.159	11.922	0.001	1.731	1.268	2.365
Constant	−5.070	0.469	116.759	<0.001			

Abbreviations: CI—confidence interval; OR—odds ratio; S.E.—standard error.

**Table 11:** Multivariable logistic regression model for patients with AKI.

Variable	B	S.E.	Wald	*p*	OR	95% CI for OR	
						Lower	Upper
Myoglobin	0.010	0.003	10.179	0.001	1.010	1.004	1.016
Hemoglobin	−0.980	0.251	15.289	<0.001	0.375	0.229	0.613
Duration of corticosteroid therapy	0.092	0.044	4.461	0.035	1.096	1.007	1.194
Constant	7.122	2.856	6.219				

Abbreviations: CI—confidence interval; OR—odds ratio; S.E.—standard error.

## Data Availability

The data supporting the conclusions of this article will be made available by the authors on request.
